# What does IGRA testing add to the diagnosis of ocular tuberculosis? A Bayesian latent class analysis

**DOI:** 10.1186/s12886-017-0597-x

**Published:** 2017-12-08

**Authors:** Rupesh Agrawal, Robert Grant, Bhaskar Gupta, Dinesh Visva Gunasekeran, Julio J. Gonzalez-Lopez, Peter K. F. Addison, Mark Westcott, Carlos E. Pavesio

**Affiliations:** 1 Moorfields Eye Hospital, NHS Foundation Trust, London, UK; 2 Biomedical Research Centre, UCL Institute of Ophthalmology, London, UK; 3 National Healthcare Group Eye Institute, Tan Tock Seng Hospital, Tan Tock Seng, Singapore; 4 St George’s, University of London & Kingston University, London, UK; 5 Royal Berkshire Hospital NHS Foundation Trust, Reading, UK

**Keywords:** Extrapulmonary latent TB, Presumed ocular tuberculosis, Positive QFT, ATT, Uveitis, Bayesian latent class analysis

## Abstract

**Background:**

To evaluate the contribution made to the diagnostic work-up for patients with suspected ocular tuberculosis (TB) by QuantiFERON-TB Gold In-Tube (QFT) tests using latent class analysis model.

**Methods:**

A single centre retrospective cohort study. A Bayesian latent class model was constructed on the basis of demographics, phenotypes and test results from patients attending a tertiary referral center in the UK. This estimated the probability of ocular TB for each patient in two versions, first with and then without QFT. The estimated probability of ocular TB was compared with treatment failure.

**Results:**

From a database of 365 patients with clinical signs suggestive of ocular TB, 267 patients who had QFT and complete data were evaluated. Mean age was 45.0 ± 15.4 years with 141 (52.9%) male and 148 (50.5%) of Asian ethnicity. QFT was positive in 208 (70.1%) patients and ATT was instituted in 145 (49.5%) patients with 100 (34.1%) patients also having concurrent systemic corticosteroid therapy. The best estimate of a QFT level separating TB-positive and TB-negative patients was extremely low. This weak discrimination between TB and non-TB groups was reflected in poor positive and negative predictive values for treatment failure.

**Conclusions:**

The latent class model did not successfully predict treatment failure, despite taking all variables into account. The threshold between TB and non-TB in QFT values was implausibly low and removing QFT from the model made prediction slightly worse. A larger prospective study is required to establish the role of all tests, demographics and phenotypes in diagnosis.

**Electronic supplementary material:**

The online version of this article (10.1186/s12886-017-0597-x) contains supplementary material, which is available to authorized users.

## Background

Tuberculosis (TB) still remains a major public health problem in most countries, including developed nations. With a large number of immigrants in the United Kingdom (UK), TB is disproportionately distributed amongst new migrants and certain ethnic groups who carry social risk factors and tend to be more adversely affected. [1] Seven thousand, eight hundred and ninety-two cases of TB were notified in the UK in 2013 with an incidence of 12.3/100,000. London accounted for the highest proportion of cases in the UK (37.8%, 2985/7892), with a rate of 35.5/100,000. [1] A significant proportion of these patients presents with extrapulmonary latent mycobacterium TB. Over the last decade, we have witnessed an apparent increase in the incidence of ocular TB. [1] This apparent increase may be attributable to a higher rate of diagnosis due to availability of the interferon gamma release assay test (IGRA). Ocular TB, as a form of latent extrapulmonary TB is faced with the multifaceted problem of diagnosis from inconclusive signs and corraborative investigations. It can present as anterior uveitis, intermediate uveitis, vitritis, retinal vasculitis, neuroretinitis, solitary or multiple choroidal tubercles, serpiginous-like choroiditis, subretinal abscess, endophthalmitis and panophthalmitis [2–6].

Ocular TB has been postulated to arise either as a result of direct infection with TB bacilli or as a hypersensitivity reaction to latent TB infection. [4, 6–8]. In-vitro assessment of interferon-gamma (IFNγ) via IGRA has been an important addition to the diagnosis of TB and has increased the sensitivity in diagnosing latent TB. [9, 10] QuantiFERON-TB Gold In-tube test (QFT; Cellestis Limited, Carnegie, Victoria, Australia) is Food and Drug Administration (FDA) approved IGRA test used for identifying latent TB infection. [9–11] According to the manufacturer’s instructions, QFT is considered positive for values > 0.35 IU/mL. Based on Centers for disease control and prevention (CDC), QFT was approved to be used for diagnosis of latent TB globally and same was adopted by National Institute for Health and Care Excellence (NICE) in the UK. [12].

The absolute values of QFT was interrogated by Gineys et al. and values of 2 IU/ml was suggested to be associated with better outcome in patients with ocular TB from low endemic settings. [13] Positive QFT results can occur in patients with non uveitic entities (optic neuritis/ orbital inflammation/ myositis/ sarcoidosis) and also in patients with non tuberculous bacilli (M. Kansasii, M. szulgai, *M. marinum*) or even on false handling of samples (with erroneously large number of TB antigens in QFT tube) leading to lack of specificity of the test. [14, 15] The reported specificity of the QFT for pulmonary and latent TB ranges from 91 to 99%, but reported sensitivity is somewhat lower 89–91%. [16, 17] The accuracy of IGRA in patients with ocular TB in a BCG-vaccinated, non-endemic population was recently evaluated and reported sensitivity and specificity were 80 and 85%, signifying impact of IGRA in this cohort of population. [18] In a recent review published by Getahun et al., the authors have looked into the worldwide literature and concluded that there is no perfect method for the diagnosis of latent mycobacterial infection [19].

The term “presumed ocular TB” is more commonly used, where no confirmatory ocular investigation is available, but the overall clinical picture, be it via systemic investigations or response to anti-tubercular therapy (ATT), makes the presumptive diagnosis of ocular TB likely. Given the lack of confirmation of local infection, this brings us to the fundamental question of whether a “presumed ocular TB” is truly an ocular TB, and which investigations are sensitive and specific enough to aid in improving the accuracy of our diagnosis. Also, the presence of a significantly broad range of positive values coupled with a myriad of clinical presentations has raised some critical questions:

- Is presumed ocular TB truly ocular TB?
- Is there any association between level of QFT positivity and likelihood of response to ATT [13].
- Do QFT values associate with any pre-treatment clinical features that might predict response to ATT in cases of suspected ocular TB, and if so, is there a threshold value? [13]

Rosenbaum et al. has described in their study on utility of routine screening of patients with uveitis for TB, post-test probability of a test can be deduced, via Bayes’ theorem, by using the known sensitivity and specificity of test as well as pre-test probability of the presence of ocular TB. [20] In the present study, we will investigate the predictive value of the QFT for the diagnosis of presumed ocular TB using a Bayesian latent class analysis of the diverse population in a tertiary eye care setting, so as to determine if QFT is justifiable as a routine screening tool for patients with presumed ocular TB. Our aims were to combine all available data into a statistical model predicting each patient’s ocular TB status, and to compare the ability of this model, with and without the QFT results, to predict treatment failure (steroid taper, defined below).

## Methods

This was a retrospective cohort study of patients seen at a large tertiary referral centre in the UK. Medical records of patients with presumed ocular TB were reviewed following approval by the Institutional Review Board (IRB). Patient records/information was anonymized and de-identified prior to analysis. This research was performed in adherence to the tenets of the declaration of Helsinki. Records of all the cases between 2005 to 2013 who had IGRA test and Mantoux test done were identified based on data from clinical laboratory at the centre.

The clinical case definition of presumed ocular TB was based on broad non-specific clinical signs proposed by Gupta et al. in 2007. [4] Clinical phenotypes suggestive of TB include anterior uveitis (granulomatous, non-granulomatous), intermediate uveitis, posterior uveitis (choroidal tubercle or tuberculoma, subretinal abscess, serpiginous-like choroiditis), panuveitis, retinitis, retinal vasculitis and neuroretinitis. Patient data were retrieved and data collected included information on demographics, morphological diagnosis, investigations and therapeutic regimens. Patients at presentation were examined by the residents, along with senior uveitis specialists, for signs suggestive of ocular TB. All patients underwent comprehensive examination and investigations to exclude other infectious diseases and immunologic disorders, including chest radiograph. Angiotensin converting enzyme (ACE) and serology tests (HIV, syphilis, hepatitis B and C) were done, if deemed necessary. Patients with a clinical diagnosis of uveitis suggestive of TB in one or both eyes were included in the study. The QFT assay was performed as per manufacturer’s recommendations and results were interpreted as per the recommendations from manufacturer. [21] Patients with one of the ocular phenotypes mentioned above, and an elevated QFT or other features suggestive of ocular TB, were referred to a respiratory physician for initiation of ATT. All attempts were made to exclude cases with sarcoidosis using information from combination of biochemical test or radiological test.

ATT was not considered in:

- Patients who had previously completed a full course of ATT within the last 10 years as recurrence in these cases was considered improbable.^10^ In this context, a positive QFT result was considered as merely immunologic memory. The authors relied on the history as notified by the patient or patient medical summary from their general practitioners, if available. No separate effort was made to collect past medical history.
- Patients who had mild anterior uveitis or retinal vasculitis, without visual loss. This is because of the frequency of unacceptable severe adverse effects of ATT as compared to the expected potential of ocular benefit [10].

Moreover, patients with insufficient follow-up visits were excluded from the study. Data were subsequently double-entered into a database by two co-authors (RA and JC) and cross-checked for accuracy; discrepancies resolved by referring back to medical records.

### Definition of outcomes

Treatment failure was defined in patients on ATT as:

- Inability to taper oral corticosteroids to less than 10 mg/ day or topical steroids to less than two times per day,
- Inability to stop oral immunosuppressive agent, or
- Persistence or recurrence of inflammation within first 6 months of completion of ATT.

For patients not on ATT, failure was defined as: (1) or (2) as stated above respectively, or: persistence or recurrence of inflammation despite treatment for at least 12 months.

### Statistical analysis

In line with manufacturer’s recommendations, values of QFT < 0.35 units were regarded as being equivalent to zero. A transformation of the QFT data was sought to facilitate model a mixture of small, positively skewed values (representing TB-negative patients) and larger, symmetrically-distributed values (representing TB-positive patients) [22].

Exploratory analyses were conducted to find differences in the use of ATT and treatment failure, as well as QFT or ACE between ethnic groups, gender, or presence/absence of binary signs, using non-parametric statistical tests. Correlation between age and QFT, as well as ACE and QFT, was assessed using Spearman’s rho coefficient. All exploratory analyses were conducted in R software (version 3.2.3) [www.r-project.org]. Raised ACE may signify a sarcoid kind of presentation and as per our recently published classification. [23] It represents tuberculous-sarcoid kind of presentation and not purely a tubercular type of presentation [23].

**Fig. 1:**
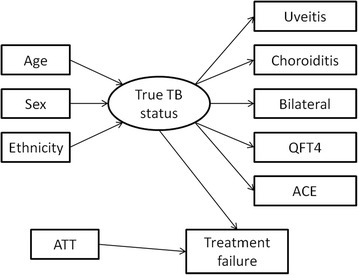
Latent model of presumed ocular tuberculosis with all possible associations. *Abbreviations:* ***TB*** *– Tuberculosis,* ***ATT*** *– Antitubercular therapy*

A Bayesian latent class model was then constructed using Stan software (rstan interface version 2.8.0) [mc-stan.org]; code is given in the technical appendix (Additional file 1). Bayesian methods are useful for statistical modelling in the context of our data because they can accommodate complex models and provide intuitive outputs regarding values of interest in terms of probability. They are solved by computer-intensive simulation to find values that fit the observed data. [24] (Additional file 1). Statistically, the estimated patient status is called a latent class, but to avoid confusion between latent class and latent TB infection, we use the term “estimated probability of ocular TB”. All associations in the model are shown together in Fig. 1. The latent class approach allows for relationships that both predict the estimated probability of ocular TB (for example, ethnicity is a proxy risk factor for exposure to Mycobacterium Tuberculosis), and are a result of, ocular TB (for example, the risk of choroiditis might differ between the patients who truly have ocular TB and those who do not). For each relationship there was a regression coefficient, and all the relationships are linked and evaluated simultaneously, along with each patient’s estimated probability of ocular TB. This approach is increasingly popular to evaluate diagnostic tests in the absence of a gold standard [25, 26].

We did not include subjective opinions as prior probabilities, but instead used the weakly informative prior approach advocated by Gelman and colleagues, [26] which avoids extreme values that are implausible and potentially problematic for computation. Residual QFT values (the mean of the observed QFT minus predicted QFT, across all steps from Stan) were plotted against observed values and other potential predictors such as chest X-ray evidence of prior active pulmonary TB to diagnose potential problems in the models. Each patient’s estimated probability of ocular TB was plotted with its confidence interval and against the recorded level of QFT to highlight strengths and weaknesses of the model. The dataset is provided in Additional file 2.

## Results

Out of a total of 365 patients with clinical signs suggestive of ocular TB, 298 patients had QFT tests, of whom 267 had complete data in all required variables for the Bayesian model. Mean age was 45.0 years with standard deviation 15.3, 141 (52.8%) were male, 141 (52.8%) were of Asian ethnicity, 55 (20.6%) African and 71 (26.6%) Caucasian. QFT was recorded as zero for 30 patients and as “< 0.35” for another six, who were counted as zero for this analysis; the mean QFT was 5.1 with standard deviation 6.3 (median 2.0, inter-quartile range (IQR) 0.1 to 8.8). Both eyes were involved in 162 (60.7%) patients. There was presence of anterior uveitis in 49 (18.4%) patients, intermediate in 65 (24.3%) patients, posterior in 95 (35.6%) patients. Choroidal involvement was present in 34 (12.7%) patients. 58 (21.7%) patients had uveitis at more than one locations with features either suggestive of panuveitis or retinal vasculitis and/or choroiditis with vitritis and anterior chamber reaction. Evidence of signs suggestive of previous pulmonary disease was present on chest X-ray for 51 patients (19.1%). The mean ACE was 37.5 with standard deviation 27.9 (median 31, IQR 22 to 44). ATT was instituted in 130 (48.7%) patients with 91 (34.1%) patients having concurrent systemic corticosteroid therapy. ATT was most commonly given for 6 months (36.2%, 47/130) or 12 months (33.84%, 44/130). Descriptive statistics are shown in Table 1.

**Table 1:** ATT use and treatment failure

	ATT given	ATT and tx failure	No ATT and tx failure
Male	61.7% (87/141)	16.1% (14/87)	29.6% (16/54)
Female	34.1% (43/126)	34.9% (15/43)	36.1% (30/83)
Asian	50.4% (71/141)	23.9% (17/71)	31.4% (22/70)
African	49.1% (27/55)	18.5% (5/27)	50.0% (14/28)
Caucasian	45.1% (32/71)	21.9% (7/32)	25.6% (10/39)
Oral steroids	60.7% (91/150)	26.4% (24/91)	50.8% (30/59)
No oral steroids	33.3% (39/117)	12.8% (5/39)	20.5% (16/78)
No choroidal involvement	48.5% (113/233)	22.1% (25/113)	32.5% (39/120)
Choroidal involvement	50.0% (17/34)	23.5% (4/17)	41.2% (7/17)
Anterior uveitis	44.9% (22/49)	22.7% (5/22)	33.3% (9/27)
Intermediate uveitis	44.6% (29/65)	24.1% (7/29)	27.8% (10/36)
Posterior uveitis	52.6% (50/95)	20.0% (10/50)	51.1% (23/45)
Posterior uveitis with choroidal involvement	47.4% (9/19)	11.1% (1/9)	60.0% (6/10)
Unilateral involvement	50.5% (53/105)	18.9% (10/53)	34.6% (18/52)
Bilateral involvement	47.5% (77/162)	24.7% (19/77)	32.9% (28/85)
QFT ≤ 0.35	46.1% (35/76)	31.4% (11/35)	24.4% (10/41)
QFT 0.35–2.00	47.4% (27/57)	25.9% (7/27)	43.3% (13/30)
QFT > 2.00	50.7% (68/134)	16.2% (11/68)	34.8% (23/66)
All patients	48.7% (130/267)	22.3% (29/130)	33.6% (46/137)

*QFT* QuantiFERON -TB Gold In-Tube test, *ATT* Antitubercular therapy

The 58 (21.7%) patients with panuveitis features (with uveitis at more than one location) appeared differed significantly from those with uveitis in the following ways:

- more of them had QFT values over the manufacturer’s threshold of 0.35 (48/58 [82.8%] compared to 143/209 [68.4%], *p* = 0.048 by chi-squared test)
- they are younger (median 40 compared to 44, *p* = 0.048 by Wilcoxon-Mann-Whitney test)
- more of them had choroiditis (15/58 [25.9%] compared to 19/209 [9.1%], *p* = 0.002 by chi-squared test)
- they had higher ACE levels (median 34 compared to 30, *p* = 0.02 by Wilcoxon-Mann-Whitney test)
- fewer of them had bilateral symptoms (27/58 [46.6%] compared to 135/209 [64.6%], *p* = 0.02 by chi-squared test)

Exploratory analyses revealed that QFT values were significantly lower in patients of Caucasian ethnicity (*p* = 0.0007), in patients not receiving oral steroids (*p* = 0.03), patients with choroiditis (*p* = 0.04), and in the presence of chest X-ray suggestive of healed pulmonary disease (*p* = 0.04). Levels of ACE (Table 1) was higher in patients with choroidal involvement (mean 45.8, median 38.5) than those without (mean 36.3, median 31.0), with *p* = 0.01. In Table 2, we show the use of ATT and the proportion with treatment failure broken down by characteristics. The striking feature is that treatment failure is higher in those not treated with ATT than those treated, for all categories except the very lowest values of QFT, which suggests that there are some false negatives in the diagnosis. We cannot estimate the false positives with the information available.

**Table 2:** QuantiFERON -TB Gold In-Tube test (QFT) values obtained in different groups of patients

Characteristics	Categories	Mean (SD)	Median (IQR)
Sex	Male (*n* = 141)	5.66 (6.65)	2.62 (0.58–9.05)
	Female (*n* = 126)	4.55 (5.94)	1.54 (0.04–7.86)
Ethnicity	Asian (*n* = 141)	5.50 (5.89)	2.65 (0.79–9.05)
	African (*n* = 55)	5.76 (6.54)	3.19 (0.08–10.40)
	Caucasian (*n* = 71)	3.92 (6.94)	0.70 (0.01–4.45)
Oral steroids	No (*n* = 117)	4.61 (6.54)	1.49 (0.02–8.00)
	Yes (*n* = 150)	5.55 (6.17)	2.47 (0.59–9.86)
Choroiditis	Absent (*n* = 233)	4.98 (6.35)	1.72 (0.05–8.76)
	Present (*n* = 34)	6.22 (6.22)	4.01 (1.65–8.86)
Uveitis	Anterior (*n* = 49)	4.83 (5.93)	1.39 (0.10–8.89)
	Intermediate (*n* = 65)	5.61 (7.50)	1.62 (0.03–10.01)
	Posterior (*n* = 95)	4.84 (5.96)	1.84 (0.05–8.26)
Bilaterality	Unilateral (*n* = 105)	4.83 (6.10)	1.51 (0.27–8.57)
	Bilateral (*n* = 162)	5.34 (6.50)	2.59 (0.04–8.90)
Chest X-ray suggestive of healed pulmonary TB	Absent (*n* = 216)	5.39 (6.53)	2.30 (0.25–8.90)
	Present (*n* = 50)	3.90 (5.32)	1.11 (0.01–6.35)
Total (*n* = 267)		5.14 (6.34)	2.01 (0.10–8.76)

*SD* Standard deviation, IQR Interquartile range

**Fig. 2:**
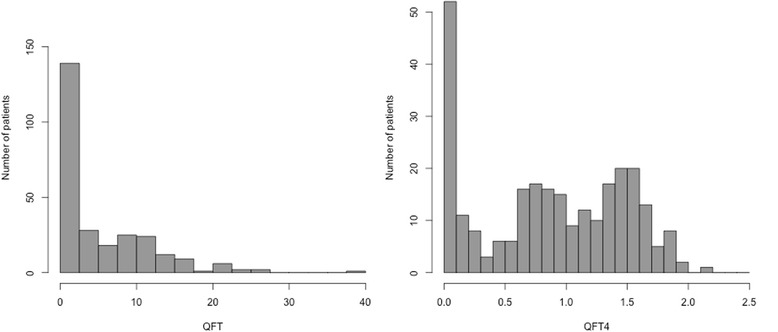
Histograms of QFT values (left) and QFT4 transformed by raising QFT to the power of 0.25 (right). *Abbreviations:* ***QFT*** *– QuantiFERON Gold In-Tube*

Raising QFT to the power of 0.25 (the ‘quartic root’) produced a distribution amenable to further statistical modelling (Fig. 2); we refer to this as QFT4. It can be seen to fall into two parts, one positively skewed with mode close to zero, and the other symmetric and centred near 1.2 (QFT = 5.28). The higher part appears bimodal (has two peaks) in this chart but with relatively small numbers, and in the absence of a biological explanation, we regard this as one common group of patients. Future research with larger samples can confirm the distribution of QFT values in this population. The least common region, which separates these two distributions and is the best candidate for a clinical cut-off level, is near 0.4 (QFT = 0.25).

Relationships between the data and the estimated probability of ocular TB are shown in Table 3 **.** This table shows, firstly, the effect of the patient characteristics on the estimated probability of ocular TB. The uncertainty in these values is represented by the wide confidence intervals (CI), and there are no significant associations, although the direction of effect is in agreement with Table 2. Secondly, the effect of the estimated probability of ocular TB on various signs and symptoms is shown, where the coefficients either represent mean differences or log-odds ratios. Posterior uveitis was significantly more common in patients likely to have TB, while anterior and intermediate uveitis was significantly less common. There was no significant difference in bilateral versus unilateral involvement. Effects on ACE and choroiditis were very imprecise, most likely for reasons of uncertainty addressed below. Finally, the effect of ATT on treatment failure is shown, and although ATT’s effect in the absence of TB is not significant, there is a significant reduction in treatment failure when TB is present, as defined by an estimated probability of ocular TB over 50%. All the patients who had previous ATT (*n* = 8) were assessed by a respiratory physician, if there was a risk of reinfection in this high-risk population. However, in absence of the retreatment, they did not demonstrated any higher risk of relapse or treatment failure. Thirty eight patients with mild or non sight threatening disease did not had ATT based on discretion of treating ophthalmologist and respiratory physician.

**Table 3:** Parameters from latent class model

	Estimated effect	95% CI
Equation estimating the probability of ocular TB		
Asian ethnicity^a^	1.33	0.94 to 1.89
African ethnicity^a^	1.13	0.76 to 1.68
Female^a^	0.89	0.64 to 1.24
Age (10 year increment, centred on 40)^a^	1.08	0.87 to 1.37
Equations predicting observed signs and symptoms based on the probability of ocular TB		
Mean QFT4 if TB+	1.19	1.12 to 1.26
*Mean QFT if TB+*	*5.15*	*4.30 to 6.19*
SD of QFT4 if TB+	0.44	0.39 to 0.49
SD of QFT4 if TB-	0.06	0.05 to 0.07
Crossover between TB+ and TB- QFT distribution (not transformed to QFT4)	0.05	0.04 to 0.07
Effect of estimated probability of ocular TB on anterior uveitis symptoms^b^	0.69	0.45 to 1.02
Effect of estimated probability of ocular TB on intermediate uveitis symptoms^b^	0.55	0.36 to 0.82
Effect of estimated probability of ocular TB on posterior uveitis symptoms^b^	1.54	1.09 to 2.18
Effect of estimated probability of ocular TB on choroiditis^b^	21,246	0.97 to 3.10
Effect of estimated probability of ocular TB on bilateral symptoms^b^	1.04	0.75 to 1.46
Effect of estimated probability of ocular TB on ACE^c^	3.99	−0.40 to 8.23

*TB* Tuberculosis, *QFT* QuantiFERON Gold In-Tube, *ATT* Anti-tubercular therapy, *SD* Standard deviation, *CI* Confidence interval

^a^This can be interpreted as an odds ratio for this characteristic in patients with tuberculous uveitis compared to those without

^b^This can be interpreted as the odds ratio for having a particular sign or symptom if the patient has tuberculous uveitis (predicted probability > 50%), compared to not

^c^This can be interpreted as an additional odds ratio that is applied to the prediction of treatment failure

**Fig. 3:**
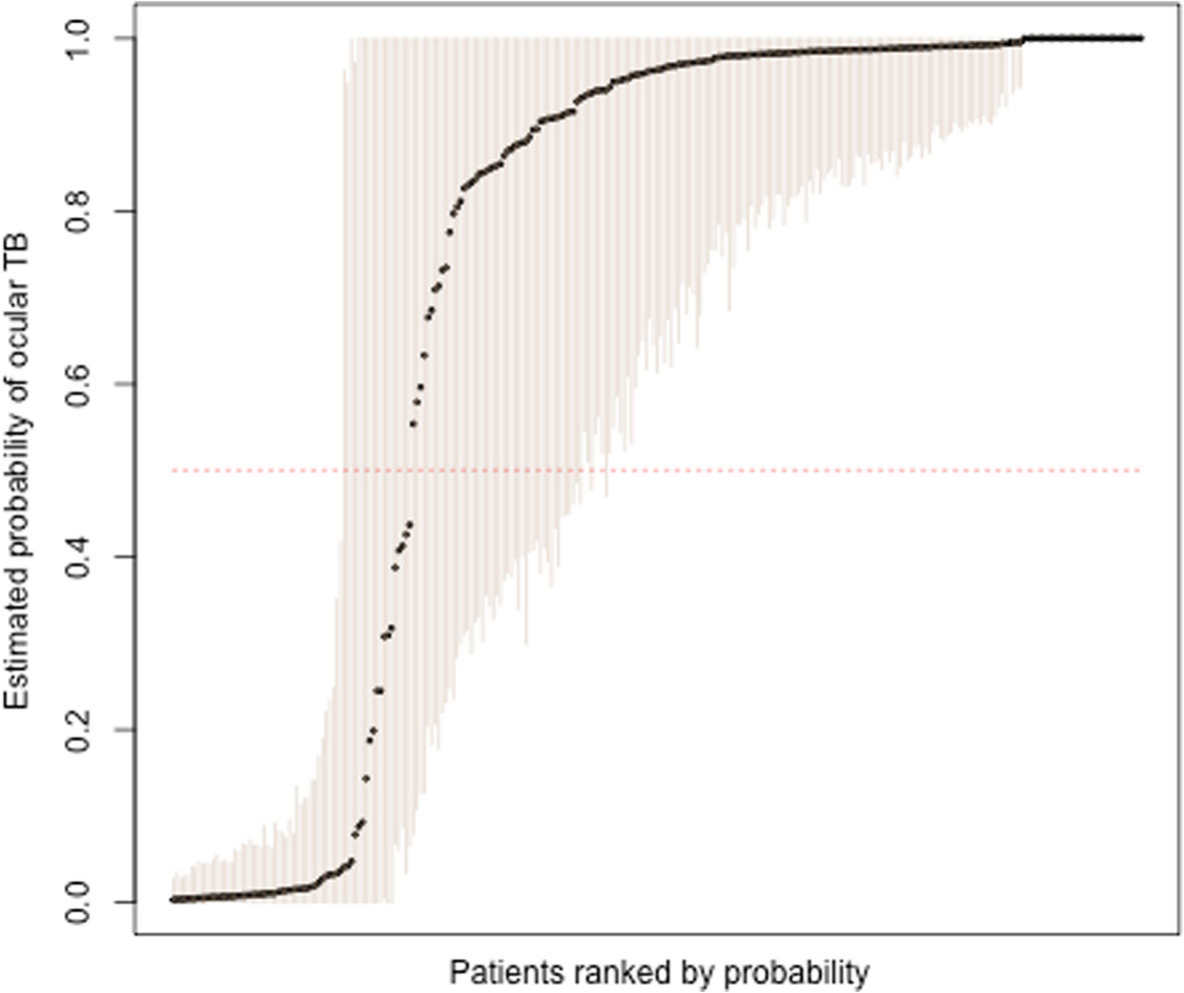
Patients’ estimated probabilities of ocular TB (black dots), ranked from lowest to highest, with 95% confidence intervals (faint vertical lines). 50% probability is shown as a horizontal dotted line

The model does not provide satisfactory predictive ability. Each patient’s estimated probability of ocular TB, with 95% confidence intervals, is shown in Fig. 3 **.** Of the 267 patients, 68 had CIs straddling either side of 0.5, while 47 were entirely below and 152 entirely above. Based on the estimated probability, taking 0.5 as a cutoff, 68 were below 0.5 and 199 above, which might be regarded as probably not TB and probably TB respectively. However, this classification corresponds poorly to treatment failure, which we expected in those who had TB and were not treated with ATT, or did not have TB but were treated with ATT. The positive predictive value (PPV) was 58.7% and the negative predictive value 56.1%, which is not much better than chance.

When the model is run again without QFT, the confidence intervals of Fig. 3 are much wider, with 259 patients straddling 0.5, 8 with CIs entirely below, and none entirely above. The estimated probability was above 0.5 for 148 patients and below for 119, and from this prediction, the PPV was 51.4% and NPV 40.3%.

**Fig. 4:**
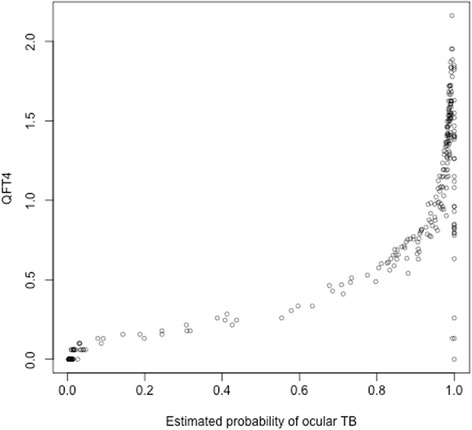
Patients’ estimated probabilities of ocular TB, plotted against the observed QFT4. A good predictive association would show as clusters of dots in the top right and bottom left parts of the plot

Despite the means of the two-part distribution being well represented by the estimates in Table 3 **,** the standard deviations are very wide. The estimated point of crossover between the distribution of TB-negative and -positive patients was at QFT = 0.05 with 95% CI 0.04 to 0.07. However, as seen in Fig. 4, the relationship between QFT4 and the estimated probability of ocular TB (which is informed in part by QFT4 itself) breaks down at high probabilities.

## Discussion

Currently there is no clear consensus on the diagnosis of ocular TB and this remains a challenge for clinicians around the world. There are many postulations behind the pathophysiology of ocular TB. It may be an immune mediated ocular inflammation that is initially triggered by mycobacterium TB, without any underlying active infection. [27] Alternatively, it may result from an inoculum directly within the eye. [28] Although such direct TB infection may manifest such as a choroidal abscess, the majority of patients displayed clinical features, which were not exclusively associated with TB infection. None of the clinical phenotypes in our series from the low endemic region showed a statistically significant association for reduced risk of recurrence with positive or better response to ATT. In our current study, we have investigated the possible predictive value of QFT for the diagnosis of presumed ocular TB from a cohort of patients with signs strongly suggestive of ocular TB, based on Bayesian model analysis.

The best estimate of a QFT level separating TB-positive and TB-negative patients is extremely low, leading most likely to high sensitivity but poor specificity. Although it is not inconsistent with the least common region seen in the observed data around 0.23, the uncertainty is too great to permit a firm conclusion separating the patients into two groups. This indicates the considerable uncertainty about true TB status, even taking all variables into account in a predictive model. In our current model, patients with higher QFT levels and treated with ATT had lower treatment failure as against those patients who did not had ATT with lower QFT values.

Ang et al. have earlier researched the usefulness of IGRA in diagnosis of presumed ocular TB, however the authors have not specified the clinical phenotype associated with positive IGRA and have instead enlisted the anatomic location of uveitis [17]. They have reported a poor sensitivity (36%) and modest specificity (75%) of IGRA test alone in presumed tuberculous uveitis; the area under the receiver-operator curve (AUC: a measure of diagnostic ability combining sensitivity and specificity that ranges from 0 to 1) of 0.555. Combining the IGRA with the results of tuberculin skin testing improved the performance somewhat (AUC of 0.644). The authors concluded that both IGRA and tuberculin skin test had poor sensitivities and that neither test could reliably exclude ocular TB if there were highly suggestive clinical features [17].

Rather than assume a gold standard and use ROC analysis, we have taken a Bayesian approach, as this allows us to express uncertainty around each patient’s estimated probability of ocular TB. Our data suggest that QFT alone does not adequately separate TB uveitis from non-TB uveitis. There are very limited studies on the possible predictive clinical signs from low endemic regions, further compounding the diagnostic conundrum. The challenge may be considered greater in countries such as the UK in which TB is much less prevalent but have a very high number of immigrant population from high endemic zones.

La Distia Nora et al. evaluated the clinical manifestations of patients with intraocular inflammation with positive QFT in nonendemic settings. [29] In the series of 77 patients, 85% of patients were found to be immigrants from endemic region. Retinal occlusive vasculitis and serpiginous choroiditis were commonly noted phenotypes with mean QFT value of 7.5 IU/ml. Like the current study, the authors did not find any positive association between QFT positivity and clinical signs. The authors and the editorial from Pepple et al. have concluded the limited role and risk of false positivity in patients with signs suggestive of ocular TB in low endemic settings and the raised QFT may albeit represent sarcoidosis. [29, 30] To further investigate the association between TB and sarcoidosis, we did correlation analysis between potential biomarkers of sarcoidosis (serum ACE) and TB (QFT). In our study we found no statistically significant association between raised serum ACE and QFT.

In another series by Ahn et al. from Korea (low endemic setting), the authors found strong correlation of positive IGRA in presumed ocular TB. [31] They also found retinal vasculitis and posterior uveitis to be the clinical phenotypes associated with tubercular uveitis (TAU) or ocular TB. Cordero-Coma et al. established immune response prevalence of 32.25% in 31 uveitis patients from Spain with estimated sensitivity of 82% and specificity of 100% with NPV of 86% for positive QFT. [32] Mackensen et al. in 2008 and Gupta et al. in 2010 had established the association of QFT with serpiginous like choroiditis. [6, 33] Babu et al. have demonstrated the use of QFT in the diagnosis of presumed ocular TB in a prospective pilot study from a high endemic region. [34] Sensitivity of QFT was shown to be 82% with specificity of 76% for diagnosis of intraocular TB based on clinical signs suggested by Gupta et al. [6, 34] However, in patients with a definite culture or biopsy proven systemic TB, the test has shown only 58% sensitivity and 77% specificity, highlighting the limitation of QFT in diagnosing active systemic TB [34].

In a population where TB is non-endemic, such as the UK, sensitivity of IGRAs is thought not to be as good as TST. [35, 36] Furthermore, specificity of the test has also been questioned. If IGRAs have helped clinicians with a new window of opportunity to diagnose latent TB it has also caused a reasonable degree of confusion with regards to its false positive and false negative results, with particular variation in low-endemic regions. It is hence essential to review the application and utility of IGRAs in low-endemic countries such as the UK.

The use of ATT to manage ocular TB is regarded as an effective treatment for ocular TB and response to therapy can be a good surrogate for diagnosis of ocular TB. Bansal et al. showed a reduced recurrence of ocular TB following ATT, in addition to steroids. [37] ATT clears latent TB infection and subsequently protects against the manifestation of active TB in up to 90% of patients. [38] Overall it would be expected that ATT would have been effective in treating ocular TB in this study and thus preventing any recurrence. It is unlikely that treatment failure in this study group could be attributed to failure of the ATT itself, and it can be compounded due to the heterogeneity in treatment regimen, inadequate treatment length, antibiotic resistance or non-compliance with the drug regimen.

Based on our observation, it can be postulated that patients with suspected ocular TB get started on steroids early if symptoms are more severe, and for the same reason they are more likely to really have ocular TB hence higher QFT. With past history of pulmonary TB, patients are enrolled in the dataset as suspected ocular TB. As the current study was not a trial of ATT and attempting to analyse the data in terms of treatment efficacy runs the risk of obtaining falsely significant results by multiple testing and confounding by indication. We sought to explore patterns in the data and generate rather than test hypotheses. Likewise, the effect of response to therapy in patients with peripheral retinal vasculitis following panretinal laser photocoagulation could not be assessed in this series, but we have highlighted the outcome of treatment in patients with peripheral retinal vasculitis in our recently published report [39].

This study has several limitations as data was collected retrospectively and researchers have made a decision based on the facts present at their disposal, which can introduce investigator’s bias. There was no information about proportion of TB uveitis patients (amongst all the new uveitis cases seen in a given year), about how many patients had new episodes of inflammation and how many had old ongoing inflammation. There was also a significant variation between oral corticosteroids, immunosuppression and ATT treatment type and length prohibiting us from drawing definitive conclusions. The definition of treatment outcome requires further validation as there is currently no gold standard or consensus guidelines defining the treatment outcome in patients with ocular TB. Furthermore, as the study was from a single centre, it may be difficult to generalize the results of this study. We propose that future directions should include conducting prospective well-designed multicentre (between low and high endemic regions) studies to understand the true formulate and accurate global depiction global picture of presumed ocular TB. A carefully designed multicentre study with clear case definition for ocular TB, non ambigous treatment outcomes, defined follow up with standardized therapy (if not randomized control trial) can tell us more information and perhaps lead to a definite conclusion.

## Conclusions

None of the defined clinical phenotypes of ocular TB or positive QFT were associated with favourable outcome with ATT. There was no evidence for a cut-off value of QFT that successfully discriminated between the two groups of patients. Extrapulmonary mycobacterium TB manifesting in the eye remains a diagnostic conundrum.

## Additional files

Additional file 1: Technical appendix to “Does IGRA test add to the diagnosis of presumed ocular tuberculosis - A Bayesian latent class analysis”. (DOCX 52 kb)

Additional file 2: The dataset. (R 25 kb)

## Abbreviations

ACE: Angiotensin converting enzyme
ATT: Anti tubercular therapy
AUC: Area under curve
IGRA: Interferon gamma release assay
IL-2: Interleukin −2
M: Mycobacterium
NPV: Negative predictive value
PCR: Polymerase chain reaction
PPV: Positive predictive value
QFT: Quantiferon –TB gold in tube test
ROC: Receiver operating characteristics
TAU: Tubercular associated uveitis
TB: Tuberculosis
TNF α: Tumor necrosis factor
TST: Tuberculin skin test
UK: United Kingdom
