# Supplementary material for: What does IGRA testing add to the diagnosis of ocular tuberculosis? A Bayesian latent class analysis
Source: BMC Ophthalmol. 2017 Dec 8;17:245. doi: 10.1186/s12886-017-0597-x (PMC5721607; doi:10.1186/s12886-017-0597-x)
Supplement: Supplementary file 1 — Technical appendix to “Does IGRA test add to the diagnosis of presumed ocular tuberculosis - A Bayesian latent class analysis”. (DOCX 52 kb) [file 12886_2017_597_MOESM1_ESM.docx]

**Technical appendix to “Does IGRA test add to the diagnosis of presumed ocular tuberculosis - A Bayesian latent class analysis”**

The software was run for 10,000 steps from 4 'chains' with different starting values (after discarding the first 5000 for ‘warm-up’ while the distribution stabilises). At each step, we calculated the QFT value where the estimated probability of tuberculous uveitis crossed 50%. This estimated the optimal QFT threshold for diagnosis. Given our assumed model of a half-normal distribution centred on μ_1_=0 with standard deviation σ_1_ but only possible for positive values, and a normal distribution centred on μ_2_ with standard deviation σ_2_ implies a crossover point at QFT4 values equal to:


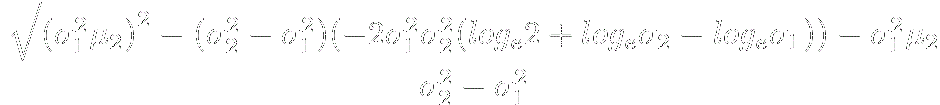


(derivation of this formula is available on request).

The Stan code for the model is given below.

data {

int N; // n with all variables known (267)

int eth1[N]; // Asian ethnicity

int eth2[N]; // African ethnicity

int female[N]; . // female

real cage[N]; // (age-40)/10 : age in ten-year units centered on 40

int failure[N]; // treatment failure

real qg4[N]; // QFT4

int au[N]; // presence of anterior uveitis

int iu[N];

int pu[N];

int ch[N];

int puch[N];

int botheyes[N];

real ace[N];

int att[N];

}

parameters {

real alpha0eth[2]; // intercepts

real alpha0fem;

real alpha0cage;

real<lower=0> cagesigma; // residual SD

real alpha_eth[2]; // association between ethnicity and prob of TB

real alpha_fem; // association between gender and prob of TB

real alpha_cage; // association between age and prob of TB

real beta0fail; // log-odds of failure if no tb and no att

real beta1fail; // log-odds of failure if tb and no att

real beta0attfail; // log-odds ratio of failure from adding att if no tb

real beta1attfail; // log-odds ratio of failure from adding att if tb

real beta0au; // intercept

real beta1au; // effect of TB status on au

real beta0iu; // intercept

real beta1iu; // effect of TB status on pu

real beta0pu; // intercept

real beta1pu; // effect of TB status on pu

real beta0puch; // intercept

real beta1puch; // effect of TB status on pu*ch

real beta0ch; // intercept

real beta1ch; // effect of TB status on choroidosis

real beta0botheyes; // intercept

real beta1botheyes; // effect of TB status on bilateral involvement

real beta0ace; // intercept

real beta1ace; // effect of TB status on ACE

real<lower=0> qgmu; // mean of TB+ qg4

real<lower=0> qgtbsigma; // SD of qg4 in TB+ cases (normal distribution)

real<lower=0> qgnosigma; // SD of qg4 in TB- cases (half-normal distribution)

real<lower=0> acesigma; // residual SD of ACE

real latentvar[N]; // latent variable

}

transformed parameters {

// latent class

real tb[N];

for (i in 1:N) {

tb[i] <- inv_logit(20.0*latentvar[i]);

}

}

model {

// declarations

real qgmean[N];

real qgsd[N];

// priors

latentvar ~ normal(0,1);

alpha0eth ~ normal(0,10);

alpha0fem ~ normal(0,10);

alpha0cage ~ normal(0,10);

cagesigma ~ uniform(0.01, 20);

alpha_eth ~ normal(0,10);

alpha_fem ~ normal(0,10);

alpha_cage ~ normal(0,10);

beta0fail ~ normal(0,10);

beta1fail ~ normal(0,10);

beta0attfail ~ normal(0,10);

beta1attfail ~ normal(0,10);

beta0au ~ normal(0,10);

beta1au ~ normal(0,10);

beta0iu ~ normal(0,10);

beta1iu ~ normal(0,10);

beta0pu ~ normal(0,10);

beta1pu ~ normal(0,10);

beta0puch ~ normal(0,10);

beta1puch ~ normal(0,10);

beta0ch ~ normal(0,10);

beta1ch ~ normal(0,10);

beta0botheyes ~ normal(0,10);

beta1botheyes ~ normal(0,10);

beta0ace ~ normal(0,10);

beta1ace ~ normal(0,10);

qgmu ~ uniform(0.05,2);

qgtbsigma ~ uniform(0.1,2);

qgnosigma ~ uniform(0.05,0.5);

acesigma ~ normal(1,5);

// likelihood

for (i in 1:N) {

eth1[i] ~ bernoulli_logit(alpha0eth[1] + alpha_eth[1]*latentvar[i]);

eth2[i] ~ bernoulli_logit(alpha0eth[2] + alpha_eth[2]*latentvar[i]);

female[i] ~ bernoulli_logit(alpha0fem + alpha_fem*latentvar[i]);

cage[i] ~ normal(alpha0cage + alpha_cage*latentvar[i], cagesigma);

failure[i] ~ bernoulli_logit((beta0fail) +

(beta1fail*latentvar[i]) +

(beta0attfail*att[i]) +

(beta1attfail*att[i]*latentvar[i]));

au[i] ~ bernoulli_logit(beta0au + beta1au*latentvar[i]);

iu[i] ~ bernoulli_logit(beta0iu + beta1iu*latentvar[i]);

pu[i] ~ bernoulli_logit(beta0pu + beta1pu*latentvar[i]);

puch[i] ~ bernoulli_logit(beta0puch + beta1puch*latentvar[i]);

ch[i] ~ bernoulli_logit(beta0ch + beta1ch*latentvar[i]);

botheyes[i] ~ bernoulli_logit(beta0botheyes +

beta1botheyes*latentvar[i]);

ace[i] ~ normal(beta0ace + beta1ace*latentvar[i],acesigma);

qgmean[i] <- tb[i]*qgmu;

qgsd[i] <- (tb[i]*qgtbsigma)+((1.0-tb[i])*qgnosigma);

qg4[i] ~ normal(qgmean[i],qgsd[i]);

}

}
